# Can we implement findings from RCTs of diagnostic tests? A review of reporting of test-treat interventions

**DOI:** 10.1186/1745-6215-16-S2-O31

**Published:** 2015-11-16

**Authors:** Lavinia Ferrante di Ruffano, Jac Dinnes, Sian Phillips-Taylor, Clare Davenport, Chris Hyde, Jon Deeks

**Affiliations:** 1 University of Birmingham, Birmingham, UK; 2 University of Exeter, Exeter, UK; 3 University of Warwick, Warwick, UK

## Objective

To evaluate the completeness of intervention descriptions in test-treatment RCTs.

## Methods

Reports of published RCTs of diagnostic tests evaluating patient outcomes published 2004-2007 were identified in CENTRAL. Two raters assessed trial reports for the presence of care pathway diagrams and for the completeness of written descriptions. Test-treat interventions descriptions were considered in their four components: 1) the test, 2) diagnosis decision-making, 3) management decision-making, and 4) interventions.

## Findings

103 trials evaluating 105 control and 119 experimental interventions were included from cardiovascular medicine (35, 34%), obstetrics and gynaecology (17, 17%), gastroenterology (14, 14%) or orthopaedics (10%, 10). Trials evaluated imaging (52, 50%), biochemical assays (25, 21%) and clinical assessment (13, 11%).

Only 3 (3%) trials mentioned all four components in descriptions of the interventions, only one of which also provided a complete care pathway diagram. Descriptions were missing or incomplete for: 42% of experimental and 71% of control tests, 57% of experimental and 71% of control diagnostic decision-making, 74% of experimental and 73% of control management planning, and 80% and 86% interventions.

## Conclusions

Reporting of test-treat interventions is very poor and worse than for other complex interventions. Current descriptions are inadequate for implementing results of these trials into clinical practice. Reporting needs to improve, with greater emphasis on describing the decision-making components of the care pathways in pragmatic as well as explanatory trials. Expansion of the TiDIER checklist for test-treat trials is required.

